# Wearable Magnetic Field Sensor with Low Detection Limit and Wide Operation Range for Electronic Skin Applications

**DOI:** 10.1002/advs.202304525

**Published:** 2023-11-30

**Authors:** Shengbin Li, Yuanzhao Wu, Waqas Asghar, Fali Li, Ye Zhang, Zidong He, Jinyun Liu, Yuwei Wang, Meiyong Liao, Jie Shang, Long Ren, Yi Du, Denys Makarov, Yiwei Liu, Run‐Wei Li

**Affiliations:** ^1^ CAS Key Laboratory of Magnetic Materials and Devices Ningbo Institute of Materials Technology and Engineering Chinese Academy of Sciences Ningbo 315201 P. R. China; ^2^ Zhejiang Province Key Laboratory of Magnetic Materials and Application Technology Ningbo Institute of Materials Technology and Engineering Chinese Academy of Sciences Ningbo 315201 P. R. China; ^3^ School of Future Technology University of Chinese Academy of Sciences Beijing 100049 P. R. China; ^4^ Mechanical Engineering Department University of Engineering and Technology Taxila Taxila 47050 Pakistan; ^5^ Center of Materials Science and Optoelectronics Engineering University of Chinese Academy of Sciences Beijing 100049 P. R. China; ^6^ National Institute for Materials Science Tsukuba Ibaraki 305‐0044 Japan; ^7^ State Key Laboratory of Advanced Technology for Materials Synthesis and Processing International School of Materials Science and Engineering Wuhan University of Technology Wuhan 430070 P. R. China; ^8^ School of Physics Beihang University Beijing 100191 P. R. China; ^9^ Institute of Ion Beam Physics and Materials Research Helmholtz‐Zentrum Dresden‐Rossendorf e.V. Bautzner Landstrasse 400 01328 Dresden Germany

**Keywords:** amorphous magnetic wires, magnetic field sensor, magnetosensitive smart skins, wearable electronics, wide detection range

## Abstract

Flexible electronic devices extended abilities of humans to perceive their environment conveniently and comfortably. Among them, flexible magnetic field sensors are crucial to detect changes in the external magnetic field. State‐of‐the‐art flexible magnetoelectronics do not exhibit low detection limit and large working range simultaneously, which limits their application potential. Herein, a flexible magnetic field sensor possessing a low detection limit of 22 nT and wide sensing range from 22 nT up to 400 mT is reported. With the detection range of seven orders of magnitude in magnetic field sensor constitutes at least one order of magnitude improvement over current flexible magnetic field sensor technologies. The sensor is designed as a cantilever beam structure accommodating a flexible permanent magnetic composite and an amorphous magnetic wire enabling sensitivity to low magnetic fields. To detect high fields, the anisotropy of the giant magnetoimpedance effect of amorphous magnetic wires to the magnetic field direction is explored. Benefiting from mechanical flexibility of sensor and its broad detection range, its application potential for smart wearables targeting geomagnetic navigation, touchless interactivity, rehabilitation appliances, and safety interfaces providing warnings of exposure to high magnetic fields are explored.

## Graphical abstract

A flexible magnetic sensor with low detection limit and wide measuring range is reported, realized by the anisotropy of the giant magneto‐impedance effect of amorphous wire. The sensor can detect the magnetic field from 22 nT to 400 mT, and simultaneously monitor weak magnetic fields such as geomagnetism and large magnetic fields which come from electromagnets etc.

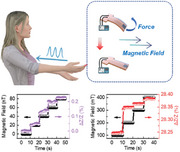

## 1 Introduction

Benefiting from their mechanical flexibility, flexible electronics can be used as components of smart wearables or conformally applied to human skin.^[^ ^1^, ^2^, ^3^, ^4^ ^]^ Human skin provides the valuable information on the surrounding environment such as temperature and pressure, which help us to perceive the world.^[^ ^2^ ^]^ Modern electronic skin (E‐skin) devices expand the abilities of our skin prescribed by nature by providing additional perception abilities like sound,^[^ ^5^ ^]^ optics,^[^ ^6^ ^]^ and direction.^[^ ^7^ ^]^ Among them, magnetosensitive E‐skins enable novel type of artificial receptors^[^ ^8^ ^]^ for sensing magnetic fields for navigation or realizing interactive human‐machine interfaces in virtual and augmented reality.^[^ ^9^, ^10^ ^]^ With the help of magnetosensitive E‐skins, we can perceive not only static but also dynamic magnetic fields.^[^ ^11^, ^12^, ^13^ ^]^

Typical magnetic fields surrounding us in our everyday life range from sub‐nT (biomagnetic fields) up to about T (field of strong permanent magnets or magnetic resonance imaging (MRI) devices). Magnetic field sensors can detect tiny magnetic fields for interactive electronics based on geomagnetic field^[^ ^7^, ^14^, ^15^ ^]^ as well as bio‐/medical applications including magnetic labeling upon cell monitoring,^[^ ^16^ ^]^ biological single magnetic bead detection,^[^ ^17^ ^]^ and multiplex protein detection.^[^ ^18^ ^]^ Strong magnetic fields can also affect the function of the brain and heart, creating potential health risks,^[^ ^19^, ^20^ ^]^ especially for patients using electronic devices such as pacemakers.^[^ ^21^ ^]^ Thus, in addition to monitoring small magnetic fields for medical and interactive appliances, the detection and early warning of strong magnetic fields is essential for human safety and security.

Contemporary wearable magnetic field sensors are designed to detect magnetic fields in specific ranges, which does not allow covering envisioned application scenarios ranging from health monitoring (detection of low fields of nT range) to safety (detection of high fields in the range of Tesla). Flexible magnetic field sensors rely on various sensing principles including magnetoresistive, magnetoimpedance, and Hall effects. Ever since the pioneering work of S. S. P. Parkin, who revolutionized the field by crafting exchange‐biased magnetic sandwiches on self‐supporting organic films, ushering in the era of flexible giant magnetoresistive (GMR) sensors with the remarkable ability to detect magnetic fields up to 4mT, this domain has attracted considerable attention.^[^ ^22^ ^]^ D. Makarov et al. successfully devised [Py/CoFe]/Cu/[CoFe/Py]/IrMn heterostructures on polyimide foils as substrates, thereby enabling magnetic field monitoring within the wide range of 2–40 mT.^[^ ^23^ ^]^ Our research team contributed by designing flexible dual spin valves on pre‐strained polydimethylsiloxane (PDMS) substrates, attaining a maximum magnetic field detection capability of 90 mT.^[^ ^24^ ^]^ A. Fert and his colleagues harnessed Co/Al_2_O_3_/Co thin films prepared on the remarkable Flexible Gel‐film platform to fabricate magnetic sensors based on the tunnel magnetoresistance (TMR) effect, capable of detecting magnetic fields up to 10 mT.^[^ ^25^ ^]^ Moreover, magnetic field‐sensitive electronic skins often employ sensors based on the anisotropic magnetoresistance (AMR) effect, owing to their intrinsic anisotropy. Notably, D. Makarov et al. successfully manufactured permalloy (Py) films and Py/Ta powder on PET and mylar substrates, respectively, attaining impressive magnetic field detection ranges of 0.2 µT–0.05 mT^[^ ^26^ ^]^ and 60 µT–400 mT.^[^ ^27^ ^]^ Sensors utilizing the magnetically driven stress effect often rely on the deformation of magnetic particles under a magnetic field to alter the material's electrical properties, X. Gong et al. ingeniously integrated Carbonyl‐iron µPs into PDMS, thereby achieving a remarkable maximum magnetic field detection capability of 150 mT.^[^ ^28^ ^]^ Similarly, D. Zhu et al. seamlessly integrated AgNWs‐Fe_3_O_4_‐PDMS and flexible organic transistors on polyimide foils, culminating in magnetic field detection ranging from 0.5 mT to 150 mT.^[^ ^29^ ^]^ Furthermore, certain materials, such as Bi, boasting non‐saturating large magnetoresistance, have the potential to profoundly enhance magnetic field detection. In a notable feat, D. Makarov et al. successfully fabricated Bismuth on polyimide foils, resulting in a magnetic field detection range spanning from 14 µT to 500 mT.^[^ ^30^ ^]^ Exploiting the Hall effect for magnetic field detection, D. Neumaier achieved an impressive maximum detection capability of 18 mT.^[^ ^31^ ^]^ Cobalt‐based amorphous wires may exhibit near‐zero magnetostriction, a feature that's beneficial in applications where changes in magnetic properties due to mechanical stress are undesirable. These wires are extensively utilized in magnetic field sensors, including giant magnetoimpedance (GMI) and fluxgate sensors. They serve as cores in many types of these sensors, especially in orthogonal fluxgates, contributing to the sensor's performance by facilitating certain magnetic properties. Co‐based amorphous alloys, from which these wires are made, exhibit good magnetic permeability and low remagnetization loss. These traits are advantageous in applications requiring efficient magnetic performance. Co‐based amorphous wires are noted for their flexibility, which could be beneficial in applications where the material's formability and ductility are important. Although it still has limitations such as high cost and poor reproducibility as a sensitive material for magnetic sensors. Compared with other materials, it has much higher sensitivity and softness than other sensors, making Co‐based amorphous wire an ideal material for preparing flexible magnetic sensors. Xiao et al. used amorphous wire to design a multimodal sensor that can detect magnetic fields as low as 50µT while being able to stretch by more than 30%.^[^ ^32^ ^]^ The fluxgate sensor based on Co‐based amorphous wire designed by Yang et al. can achieve magnetic field measurements as low as 0.1 nT.^[^ ^33^ ^]^ Compared with other materials, it has much higher sensitivity and softness than other sensors, making Co‐based amorphous wire an ideal material for preparing flexible magnetic sensors.

This survey of the recent reports on flexible magnetic field sensors reveals that it is difficult to realize low detection limits and wide operation range in a single sensing element.^[^ ^34^, ^35^, ^36^ ^]^ We note that this is not the issue specific to flexible magnetoelectronics. The same problem is discussed also for well‐established rigid magnetic field sensors. To achieve a wide range of magnetic field measurements, a typical approach is to integrate a variety of magnetic field sensors in a single device.^[^ ^37^ ^]^ However, this approach requires more complex signal conditioning circuits, which increases the complexity of the sensor system design. Therefore, realizing a single sensing unit revealing high mechanical flexibility, low detection limit, and wide operation range remains an unsolved problem of current magnetic field sensor technologies.

Herein, we report a flexible magnetic field sensor having a wide sensing range from 22 nT to 400 mT and low detection limit of 22 nT. Our sensing unit includes an amorphous Co‐based microwire revealing GMI effect, which enables detection of low magnetic fields of 22 nT while providing mechanical flexibility. The GMI wire alone is magnetically saturated in a magnetic field of less than 50 mT. To sense higher magnetic fields, we designed a cantilever beam structure consisting of a flexible permanent magnet patch and a GMI wire. In this system, the cantilever beam is bent when exposed to an external magnetic field. The change of the angle between the amorphous wire and the magnetic field results in a continuous change of the impedance of the amorphous wire providing the possibility to also measure stronger magnetic fields of up to 400 mT maintaining a low limit of detection down to 22 nT. By harnessing this novel cantilever beam structural design, we efficaciously augment the detection ambit of sensors anchored on amorphous wires, renowned for their pronounced sensitivity. This innovation ensures their adaptability across both minuscule and pronounced magnetic field contexts. The sensor is mechanically flexible and compact and operates not only when worn on skin but also in a constrained environment being integrated in a decorative fingernail. These smart magnetosensitive wearables can help us perceiving the actual magnetic fields surrounding us in our daily activities. We demonstrated the application potential of our wearable magnetic field sensors for geomagnetic navigation, realization of interactive human‐machine interfaces for entertainment and rehabilitation purposes as well as safety warning systems in case exposure to strong magnetic fields.

## 2 Results and Discussions

### 2.1 Concept of Wearable Magnetic Field Sensor

**Figure 1:**
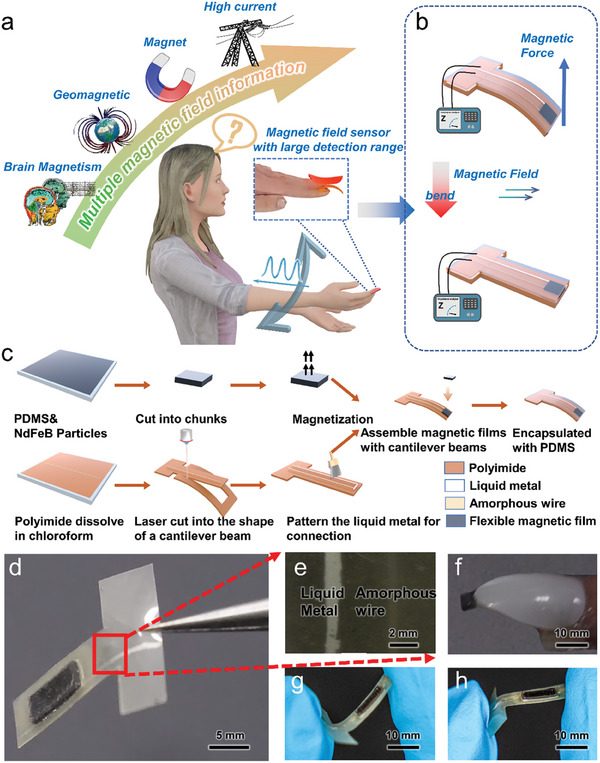
Flexible amorphous wire‐based magnetic field sensor for wide‐range magnetic field measurements and low detection limit. a) Schematic diagram of magnetic field detection. The sensor can be applied to or integrated in a decorative fingernail. The sensor is in the form of a cantilever beam and can detect magnetic field of different strength. b) Detection principle of the flexible magnetic field sensor. When the magnetic field is small, the cantilever beam retains its bent state. The magnetic field is measured based on the GMI effect of the amorphous wire itself. When the magnetic field became stronger, due to the interaction between the magnetic field and the magnetic patch, the cantilever beam and the amorphous wire are bent in the parallel direction to the magnetic field. Thus, the response curve of the amorphous wire to the magnetic field and the impedance of the sensor changes. c) The preparation process of the sensor: an elastic permanent magnet patch is formed by mixing NdFeB and PDMS. The patch is magnetized in a pulsed magnetic field and placed on a cantilever beam accommodating a GMI wire‐based sensor. Conductive lines are formed by brushing liquid metal at appropriate places for contacting the sensors. The entire device is encapsulated in PDMS. d) Optical image of the sensor and e) a close‐up of the amorphous wire and liquid metal contacts. f) The sensor is applied to a decorative nail. g,h) Bendability of the sensor devices.

Taking into account that interactivity is typically realized by pointing with our pointing finger on the object of interest or touching the screen of a smartphone, it is instructive to apply flexible magnetic field sensors to the finger (**Figure** **1**a). Figure 1b shows the measurement principle of the sensor.

When the magnetic field is small (not sufficient to bend the cantilever beam, accommodating the GMI wire, to the field direction), the sensing mechanism is based on the GMI effect of the magnetic amorphous wire. The interaction force between the magnetic field and the magnet block is very small and can be ignored. At this time, the shape of the cantilever beam itself remains unchanged. When the magnetic field increases, the amorphous magnetic wire becomes magnetically saturated. However, the interaction force between the magnetic field and the magnet block increases to the point where it is difficult to ignore. Under the influence of the magnetic force, the cantilever beam bends in the direction of the magnetic field. The angle between the amorphous wire and the magnetic field changes accordingly, causing the sensor saturation impedance to change (Figure S1, Supporting Information). Thus, we can rely on the bending of the cantilever beam in the field direction to achieve larger magnetic field detection.

In this case, the impedance of the amorphous wire may change due to the magnetic field (magnetoimpedance effect) and the stress (stress impedance effect). Both of these effects originate in the change of the magnetic domain pattern driven by an external stimulus. We confirm experimentally (Figure S2, Supporting Information) that when the amorphous wire is in a magnetically saturated state, the applied stress hardly affects its impedance. This can be understood based on the consideration that for thin amorphous wires, mechanical bending does not produce sizable stress. Therefore, if the amorphous wire is saturated in a high magnetic field, the sensing mechanism is mainly based on the anisotropy of the sensitivity of the amorphous wire to the direction of the applied magnetic field^[^ ^38^, ^39^ ^]^ (Figure S2, Supporting Information). For the Co‐based wire used in our work, there is a distinct difference in the magnetoimpedance (MI) response of the sensor to magnetic fields applied along (13 Ω) or perpendicular (15 Ω) to the wire (the impedance change differs by 8% with respect to the nominal impedance of 25 Ω).

### 2.2 Preparation of Flexible Magnetic Sensor

The fabrication process of the sensor is shown in Figure 1c, which includes the fabrication of a mechanically flexible cantilever beam structure accommodating Co‐based amorphous wire and a flexible permanent magnet. The elasticity of the cantilever allows it to undergo small displacements when a force is applied. This displacement can be detected by electronic devices and converted into electrical signals, enabling sensitive detection of external stimuli.^[^ ^40^ ^]^ This conversion is so sensitive that even very small mechanical displacements can be detected and converted into readable electrical signals.

The mechanical stability is given by the flexibility of the amorphous magnetic wire connected using a liquid metal interconnect with excellent tensile properties. Co wire and liquid metal interconnects are encapsulated in a cantilever structure made of polyimide. A flexible permanent magnet is based on hard magnetic particles (NdFeB, 9 wt%) dispersed in an elastomer matrix (PDMS) and is placed on one end of the cantilever structure. The sensor structure is encapsulated with PDMS to assure mechanical integrity and biocompatibility.

Compared to manually fabricated methods, 3D electronic printing technologies, such as inkjet or aerosol jet printing,^[^ ^41^, ^42^, ^43^ ^]^ undeniably enhance the repeatability and reliability of sensors. Nevertheless, when it comes to complex structures including sensitive materials, encapsulation materials, and conductive pathways, direct 3D printing exist numerous challenges. So, we currently use manual fabricating to prepare our sensors.

The sensor of the final cantilever structure is shown in Figure 1d. Amorphous wire and liquid metal interconnects are distributed on both sides of the cantilever beam (Figure 1e). In the initial state, the sensor cantilever is curved, as shown in Figure 1f. The entire sensing unit is compact and can be seamlessly integrated even in a decorative fingernail capable of real‐time detection of magnetic fields in a wide range. At the same time, it has good flexibility and can withstand bending in different directions (Figure 1g,h) as it is relevant for smart skin and smart textile applications of this technology.

### 2.3 Structure and Performance Characterization of the Magnetic Field Sensor

We characterized amorphous Co‐based wires of different diameters (Figures S4 and S5, Supporting Information). The morphology of the amorphous wires was observed by scanning electron microscope (SEM) and energy dispersive spectrometer (EDS), as shown in Figure S3 (Supporting Information). The wire has a smooth glass fiber package. Wires are mainly composed of Cr, Fe, and Co. The magnetic hysteresis loop of a Co‐based amorphous wire was measured by applying a magnetic field along the wire in a vibrating sample magnetometer (VSM). The hysteresis loop is almost closed, which is characteristic of a soft magnetic material (Figure S5, Supporting Information). The saturation magnetic field is about 2.5 mT. The magnetic permeability of the sensor decreases with the increase of the driving frequency of the magnetic field and then remains almost unchanged after 500 Hz. Here, 30 µm wire has the highest magnetic permeability. The GMI effect in amorphous wires has been associated with a rapid change in the skin depth, driven by the low‐field sensitivity of the azimuthal dynamic permeability. Thus, the amorphous wire with larger magnetic permeability has better GMI performance,^[^ ^32^ ^]^ and the 30 µm amorphous wire has the best performance, so we chose an amorphous wire of this size.

Furthermore, we characterized the impedance change of the amorphous wire under magnetic fields of different driving frequencies and currents. From Figure S6 (Supporting Information), it can be found that as the driving frequency increases (0.5 kHz–5 MHz), the MI effect of the amorphous wire under 1 mA driving current increases from nearly 0% to a maximum of 58%. The effect of driving current can be explained by considering the tensor character of magnetic permeability. The different dependences of GMI on driving alternating current can also be attributed to the difference in the domain structures of the investigated samples. At low‐amplitude currents, there's an inhomogeneous distribution of local critical magnetic fields, causing the “spike” feature in GMI profiles or the instability of the GMI signal. As the current increases, a corresponding magnetic field will be generated inside the amorphous wire, thereby magnetizing it, and making the local magnetic field evenly distributed. Pervious work also shows, under certain range of the currents, increasing the amplitude can greatly improve the GMI signal.^[^ ^44^ ^]^

When the amorphous wire is bent, its impedance will decrease accordingly. This is because the amorphous wire is affected by stress during the bending process. The giant stress impedance effect of amorphous wire will cause it to reduce its impedance under the action of stress (Figure S2, Supporting Information). We tested the change of amorphous wire impedance with a magnetic field under different bending conditions (Figure S7, Supporting Information). It can be seen that the maximum impedance change rate of the sensor decreases as the angle decreases. The reason for this phenomenon is that on the one hand, the decrease in the initial impedance reduces the change rate, and on the other hand, the bending of the amorphous wire also causes the magnetic field to decrease the axial component of the amorphous wire.

Then we investigated the impedance change of the amorphous wire under magnetic fields of different driving currents and frequencies. The change of the driving current will affect the initial radial magnetization of the amorphous wire, thus affecting the change of its impedance with the external magnetic field. Therefore, we explored the MI effect of the amorphous wire under the driving current varying from 0.1 to 40 mA (Figure S8, Supporting Information) with the 5 MHz driving frequency. With the increase of the driving current, the MI response first increases and then decreases revealing a maximum of 40% at 1 mA.

**Figure 2:**
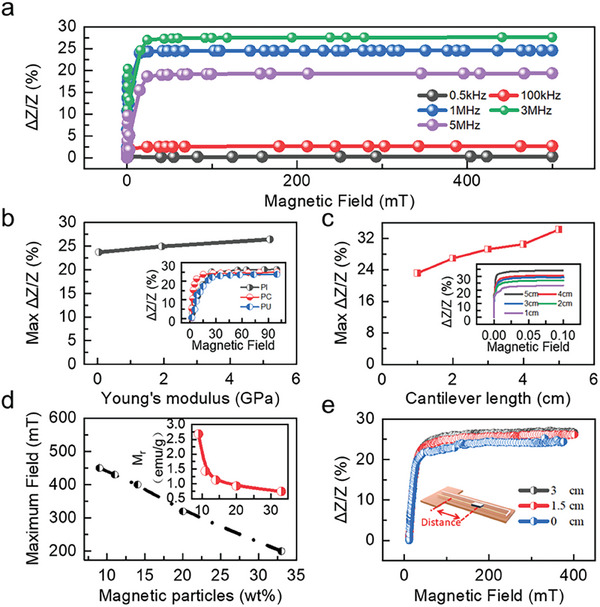
Characterization of the flexible magnetic field sensor. a) Response of the flexible magnetic field sensor to the magnetic field. The sensor is driven at different frequencies with PI substrate. The driving current is 1 mA. b) Response of the flexible magnetic field sensor to the magnetic field when the sensor is fabricated on different substrate material, with 3 MHz driving frequency and 1 mA driving current. c) Magnetic field response of sensors with prepared on cantilever beams of different length made of polyimide material. d) The maximum field, which can be detected with our sensor, when using magnetic patches with different concentration of magnetic particles. The insert shows the magnetic remanence of the composites with different content of magnetic particles. As the PDMS content increases, the remanence magnetization (M_r_) of the magnet patch decreases, while the maximum field, which can be detected by the sensor, increases. e) MI performance of the sensor dependent on the position of the magnet patch, which is located at different positions on the cantilever beam.

However, when the amorphous wire is integrated into the sensor structure with liquid metal interconnects, it exhibits a different frequency response compared to an individual amorphous wire. The MI response of the integrated sensor with polyimide substrate first increases and then decreases with frequency (**Figure** **2**a) with its maximum of 28% at 3 MHz while under the 1 mA driving current. We anticipate that the reason for this observation is related to the change of the skin effect of the connecting line, which is used to connect the sensor (Cu and liquid metal). At higher frequencies, the skin effect becomes more pronounced resulting in a higher impedance of the sensor and its weaker sensitivity to the applied magnetic field.

Similarly, the choice of the sensor substrate will affect the mechanical performance of the sensor under stress, thereby affecting the sensor's ability to detect large magnetic fields. To this end, we tested various polymer materials with different mechanical properties (polyimide (PI), polycarbonate (PC), and polyurethane (PU)) to prepare sensing devices and characterized the sensor response curves when exposed to magnetic fields. First, we obtain the stress‐strain curve of the material, thereby calculating the elastic modulus (Figure S7, Supporting Information). Among them, polyimide material has the highest Young's modulus (1.3 GPa). When it was used as a substrate for the wire‐based sensor, as shown in Figure 2b, the sensor revealed the highest MI response of 27% with the 3 MHz driving frequency and 1 mA driving current. Still, sensors prepared on other substrates also have excellent magnetic detection ability with the MI effect of 24–25%. Hence, relying on our technology, we can select the substrate with respect to its mechanical properties to match the requirement of the selected application scenario. In the following, we discuss sensors prepared on polyimide as it provides the largest operation range for our sensor.

We explored the response of cantilevers of different lengths (1–5 cm) to magnetic fields (Figure 2c). The MI effect increases from 20% to 35% as the length of the cantilever increases with the 3 MHz driving frequency and 1 mA driving current. This is because the shorter cantilever beam makes the amorphous wire closer to the magnet patch, thus having a smaller initial impedance (the magnetic field of the magnet patch itself will reduce the impedance of the amorphous wire), resulting in a smaller impedance change. Considering that wearable devices require a smaller size, we used a 3 cm‐long cantilever beam structure in subsequent experiments.

Further, we characterize the hysteresis loops of the flexible magnet patch with different PDMS and NdFeB composition (Figure S10, Supporting Information). As the content of NdFeB in the composite decreases from 33 wt% to 9 wt%, the saturation magnetization of the magnet decreases from 35.5 to 8.7 emu g^−1^. The magnetic moment of the magnet patch at the free end of the cantilever beam will affect its interaction force with the external magnetic field. In this way, the operation range of the sensor at higher fields will be affected. In particular, reduction of the saturation magnetization of the composite makes the sensor more difficult to deform and increases the sensor's measurement range (Figure 2d). For instance, having a PDMS:NdFeB composite with a 10:1 weight ratio (9% wt NdFeB), the sensor is capable of achieving magnetic field detection up to about 450 mT, while the sensor having PDMS:NdFeB composite with a 2:1 weight ratio (33% wt NdFeB) can only detect the magnetic field of about 200 mT.

The position of the magnet on the cantilever beam also affects the operation range of the sensor. We placed small patches of the flexible magnet at the top (3 cm to the tail of the cantilever), center (1.5 cm to the tail of the cantilever), and tail of the polyimide cantilever beam, and assessed the corresponding MI performance of the sensor with 3 MHz driving frequency and 1 mA driving current(Figure 2e). As the position of the flexible magnet approaches the top of the cantilever beam, the MI effect increases from 22% to 28%. This enhancement can be explained considering that the position of the magnet patch affects the torque of the magnetic field and its interaction force, thereby changing the effect of the magnetic force and affecting the detection range of the sensor. We built the following model to explain this phenomenon: Since the initial bend of the cantilever beam is 45°, the direction of *B* relative to the magnet's moment changes along the length of the beam.

The torque τ on the magnet due to the magnetic field is given by:

(1) τ=m×B

where the magnitude is given by:

(2) $$\tau \;=\;mBsin\left(\theta \right)$$

where θ is the angle between *m* and *B*.

As the location of the magnet is changed from the base toward the free end of the cantilever, the angle *θ* between the magnetic moment *m* and *B* increases from 45° to 90°.

Since $\sin\left(45^{\circ }\right)=\sqrt{2}\;/2$ and $\sin(90^{\circ })=\;1$, the torque τ on the magnet due to *B* will increase as the magnet is moved toward the free end. This means the moment exerted on the cantilever beam will also increase as the magnet is moved toward the free end.

The moment at the free end will be maximum because there the angle *θ* is maximum, that is, 90°. And the moment at the fixed end will be minimum because there the angle *θ* is minimum, that is, 45°.

In summary, the moment on the cantilever beam due to the magnet's interaction with the magnetic field will increase as the magnet is moved from the fixed end to the free end of the cantilever. The larger moment makes our cantilever beam return to the parallel state faster, which reduces the range of the sensor.

### 2.4 Magnetic Field Detection Performance of the Flexible Magnetic Field Sensor

**Figure 3:**
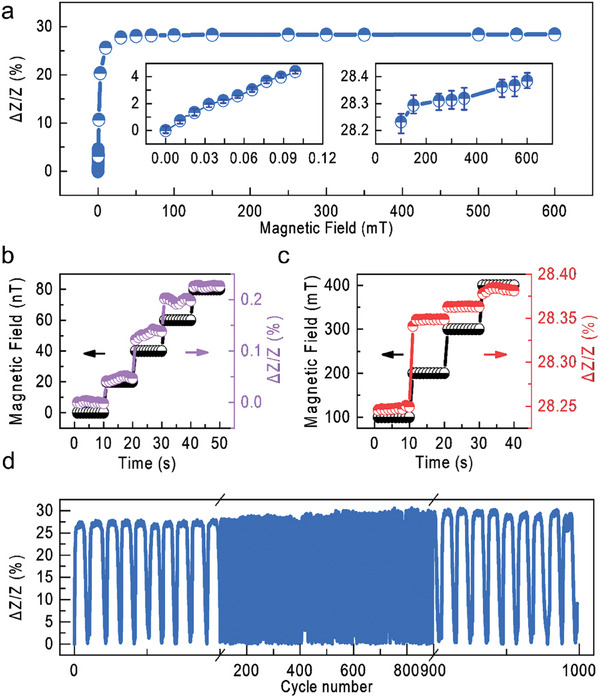
Response of the flexible magnetic field sensor to the magnetic field of different strength. a) MI response of the sensor in the range of 0 – 600 mT. Insert is an enlarged view of the sensor response at 0‐0.1 mT and 100–600 mT. b) MI performance of the sensor (right y‐axis) exposed to magnetic field, which increases 22 nT every 10 s (left y‐axis). c) MI performance of the sensor (right y‐axis) exposed to magnetic field, which increases 100 mT every 10 s (left y‐axis). d) Stability of the MI performance of the sensor exposed to 100 cycles of an applied magnetic field of 200 mT. The sensor is made of PI substrate, with a measurement frequency of 3 MHz, a current of 1 mA, a cantilever beam length of 3 cm, and a magnet at the top.

In **Figure** **3**a, we demonstrate that our sensor can respond to magnetic fields over a wide range (0–600 mT). Using Helmholtz coils setup, we increased the magnetic field by 22 nT increment every 10 s. We demonstrate that for each strength of the magnetic field, the MI effect of the sensor increases by about 0.1%. This demonstrates that we can detect small magnetic fields as low as 22 nT with our flexible sensor (Figure 3b), and the GF value can reach up to 400. At the same time, when the magnetic field increases, the deflection of the cantilever changes the angle between the amorphous wire and the magnetic field so that the sensor can detect the magnetic field exceeding its saturation magnetic field. We measured the change of the sensor impedance by increasing the magnetic field with an increment of 100 mT every 10 s (Figure 3c). It can be seen that the impedance change rate of the sensor after 200 mT is about 0.01%/100 mT, and the GF value can reach ≈0.04. Thus, our sensor has a measurable response to magnetic field range in a wide range (22 nT–400 mT). We further measured the sensor's response to the magnetic field at 0–30 nT. From Figure S11 (Supporting Information), we believe that the sensor can resolve a magnetic field of about 2 nT.

We then studied the response of the sensor under different magnetic fields. Due to the anisotropy of the amorphous wire to the horizontal magnetic field and the vertical magnetic field (Figure S2, Supporting Information), the sensor responds differently to magnetic fields at different angles. The sensor will undergo obvious periodic changes during rotation in the magnetic field, which also shows that our sensor can sense the angular changes of the magnetic field (Figure S12, Supporting Information).

In Figure 3d, we explore the long‐term stability of the sensor. We use an electromagnet to cycle a 200 mT magnetic field 1000 times and measure the sample impedance upon cycling. As the number of cycles increases, the MI effect of the sensor remains at the level of 28% without any notable degradation. Therefore, our sensor not only has excellent magnetic detection ability but also has stable repetitive detection ability.

Response time is also an important parameter of the sensor. Here we characterize the response time of the sensor after applying a 22nT magnetic field. The response time only takes 0.03 s (Figure S13, Supporting Information), which can quickly respond to changes in the magnetic field. We then prepared three identical sensors using the same manufacturing method and characterized their initial impedance and saturated impedance change rate. As shown in Figure S14 (Supporting Information), although the manual process limits the uniformity of the sensor, its impedance change rate and resistivity change little, shows that the sensor has good repeatability.

Compared with different types of previously reported flexible magnetic field sensors (Table S1, Supporting Information), our sensor has the lowest detection limit (much lower than other sensors) and can achieve magnetic field detection spanning 7 orders of magnitude while having the lowest detection limit. and the widest detection range.

### 2.5 Applications of Flexible Magnetic Field Sensors

**Figure 4:**
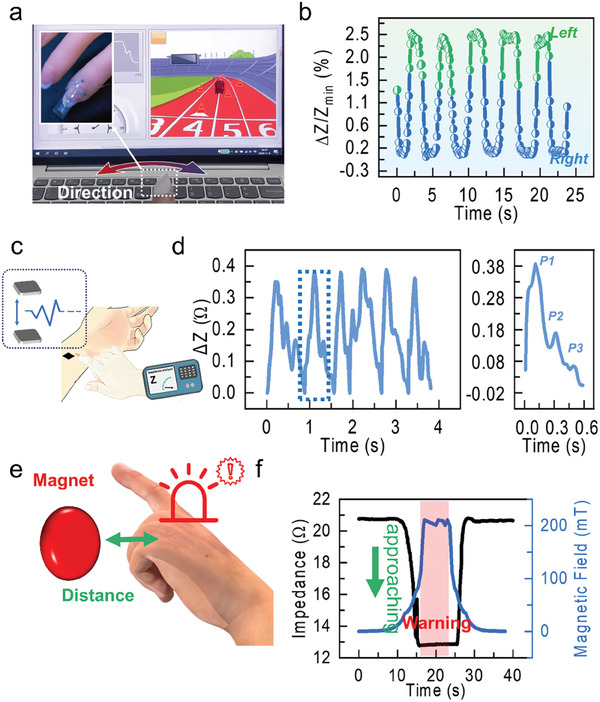
Application scenarios of wearable magnetic field sensors. a) Gaming demonstration where a wearable sensor applied to a finger nail is used to guide the direction of a car relying on the interaction with geomagnetic field (about 40 uT). The displacement of the car to the left or to the right is controlled by the swing of the hand (Video S1). The insert is an enlarged view of the sensor applied to the finger. b) The change of the impedance with the swing of the finger, which is used to control the direction of the car in the game as shown in panel (a). c‐d) The measurement of the pulse beat using the sensor. A small flexible magnet patch is applied to the arm. The sensor on a finger is brought in proximity to the magnet patch, which allows to measure the pulse caused by the change in the magnetic field due to the tiny displacement of the magnet. Typical magnetic field, which is detected by the sensor in this demonstrator is 10 mT. e‐f) Safety application demonstration. The same sensor as in previous examples can be used to detect strong magnetic fields to warn the wearer on the undesired exposure. The magnetic field threshold for this particular demonstrator is set to 200 mT (Video S2). The sensor is made of PI substrate, with a measurement frequency of 3 MHz, a current of 1 mA, a cantilever beam length of 3 cm, and a magnet at the top.

We applied our sensor to the finger (**Figure** **4**a,c) or integrated in a decorative nail (Figure 4e) to demonstrate its application in daily life. This smart wearable can help us perceiving the magnetic field of the environment in real‐time, for instance to realize geomagnetic navigation (Figure 4a, Video S1, Supporting Information). When we move the finger decorated with the sensor, the angle between the amorphous wire and the geomagnetic field changes, thus changing the impedance of the sensor 2.7% at the maximum (Figure 4b). In the process of finger movement, we measure the impedance of the sensor in real‐time and input it to computer realizing human‐machine interface for touchless interactivity. In this way, we exemplarily show the realization of the virtual reality interface for real‐time precise control of the driving direction of a car in a computer game (about 0.4° theoretical accuracy, 0.5 Ω impedance variation per 180° with 0.001 Ω accuracy of impedance analyzers). This demonstrator suggests that our sensor can be used also to interact with virtual displays for prospective augmented reality applications. This setup can be used for rehabilitation purposes to improve fine motoric function of fingers after injuries or as an interface to train concentration of participants.

For example, we attach a small, flexible magnet to the arm at a spot where the pulse can cause a slight movement. This movement occurs because the pulse creates a rhythmical throbbing, which in turn, causes the magnet to move back and forth slightly. Sensors can detect this movement since it causes a change in the magnetic field surrounding them, specifically at the location of the sensor. Now, when a smart wearable device, equipped with these sensors, is brought close to the area where the flexible magnetic patch is attached, it can successfully monitor and track the human pulse (as illustrated in Figure 4c). Furthermore, Figure 4d demonstrates how the sensor's impedance (resistance to electrical flow) changes in response to the pulse. These changes in impedance allow for the accurate identification of key peaks, labeled as P1, P2, and P3 peaks, within the pulse waveform. Through this setup, the wearable device can effectively capture and analyze the pulse, providing valuable data for health monitoring or other applications.

In Figure 4e, we demonstrate the application potential of the smart magnetosensitive wearable for safety and security applications. The increase of the sensor's impedance when approaching to a source of strong magnetic field is shown in Figure 4f. For this demonstration, we used a piece of a strong permanent magnet. When the sensor is exposed to a magnetic field of certain strength (here, stronger than 200 mT), the system is programmed to send a warning signal, thus preventing exposure to strong magnetic fields. This is crucial when the person should not be exposed to stronger magnetic fields for a long time as prescribed by the World Health Organization or for people using supporting medical equipment, for example, pacemakers. For the later, fields in the range of 10 mT are already strong enough to influence the device.^[^ ^45^ ^]^ Upon approaching an environment with high magnetic fields like a MRI instrument or high‐current carrying wires in electrical vehicles or electromagnets (Video S2, Supporting Information), our wearable sensor can be used to warn the user for a potential danger.

Although our sensor impedance measurement currently requires connecting to an external measurement device (impedance analyzer), there are already some impedance analysis devices that can be carried around. At present, people have integrated the AD5933 chip to realize an impedance analysis instrument that can be carried daily.^[^ ^46^, ^47^ ^]^ It has frequency testing capabilities from 5 Hz to 100 kHz and can measure impedance from 10 Ω to 100 kΩ. Based on the rapid development of wearable electronic devices, it is expected to realize wearable high‐precision impedance analysis equipment in the future.

We emphasize that all demonstrations discussed above from medical applications through interactive electronics up to the safety systems were done with the same wearable sensing unit, which can detect small and large magnetic fields.

## 3 Conclusion

In this work, we demonstrate a wearable magnetic field sensor with a low detection limit of 22 nT and wide operation range from 22 nT to 400 mT. Our sensor unit benefits from the giant magnetoimpedance effect and anisotropy of the magnetoimpedance response of the amorphous wire when exposed to a magnetic field of different orientation. When the magnetic field is small, we rely on the MI effect of amorphous wire to achieve high‐precision magnetic field measurement. When the magnetic field becomes larger, it will saturate the wire. Therefore, we use the cantilever beam structure to convert the change of the magnetic field into the interaction force between a patch of flexible magnet and the magnetic field. This will drive the cantilever beam to bend, thereby changing the angle between the amorphous wire and the magnetic field. Using the anisotropy of its magnetic response, the sensor can continue measuring the magnetic field after saturation. With this flexible magnetic field sensor technology, we can achieve a magnetic field measurement in the range from 22 nT to 400 mT. Based on this design, our sensor can easily detect various types of magnetic fields that appear in our everyday life. Due to the flexibility of the sensor, it can be easily applied on skin or integrated in smart wearables even as a component of a smart decorative nail. The sensor can detect the geomagnetic field so that it can be used in fields that require direction finding such as navigation and can also accurately measure the positional relationship between the sensor and a flexible permanent magnet. This can be used for rehabilitation purposes to improve fine motoric function of fingers after injuries or as an interface to train concentration of participants. We used this wearable system to accurately detect the magnetic field fluctuations of the small patch of flexible magnet on the arm during the rise and fall of the human pulse, resulting in the capturing of the pulse curve. At the same time, our sensor can also issue a warning when the external magnetic field is too large, reminding and protecting the user, especially those users wearing magnetic field‐sensitive electronic devices (such as cardiac pacemakers). Therefore, our work achieves low detection limits and wide‐range magnetic field measurements in a single device, opening exciting prospects for the application of wearable magnetic field sensing devices for interactive electronics, bioelectronics, safety, and security applications.

## 4 Experimental Section

### Preparation of Liquid Metal

High‐purity metals—Gallium, Indium, and Tin (99.99%, Beijing Founde Star Sci. & Technol. Co., Ltd) were amalgamated in a mass ratio of 68.2:21.8:10. Subsequently, the mixture was subjected to a thermal treatment at 60 °C for a duration of 30 min whilst being stirred, culminating in the formation of the LM Galinstan alloy (Ga68.2In21.8Sn10). Following this, the synthesized Galinstan was amalgamated with micron‐sized copper powder in a mass proportion of 9:1, whereupon the resultant blend underwent multiple cycles of heating and vacuum treatment to expunge air bubbles. The integration of copper powder with the liquid metal engendered a semi‐liquid metal composite (Cu‐EGaIn), thereby augmenting the wettability and adhesion characteristics.

### Preparation of Flexible Magnetic Field Sensors

The substrate of the sensor was prepared by dissolving polyimide powder (DuPont, USA) in chloroform. While for PC and PU substrates, its powder (DuPont, USA) was prepared in acetone. The resulting mixture was placed in a glass mold for 24 h to form a PI/PC/PU film with a thickness of 2 mm. The resultant mixture was placed in a glass mold for 24 h to form a film having a thickness of 2 mm. Cured polyimide film was cut into cantilever shapes, which were further used to assemble the sensor. The amorphous wires (30, 50,100 µm diameter, Aichi‐steel, Japan) and liquid metal (1.5 Ω cm^−1^) interconnects were placed at the center of the film, and the two ends of the wires were connected to the external electronics using Cu wires with diameter of 0.2 mm. Liquid metal interconnects were based on high‐purity alloys of Gallium, Indium, and Tin (99.99%, Beijing Founde Star Sci. & Technol. Co., Ltd, China), which were mixed in the ratio of 68.2:21.8:10 by mass and heated and stirred at 60 °C for 30 min. Small patches of flexible magnets were formed by mixing PDMS (184, Dow Corning, USA) and NdFeB particles (diameter: 50 µm, Xinnuode Co., Ltd, China). After curing and cutting by laser, the composites were magnetized in a pulsed magnetic field of 5 T with a pulse duration of 1 ms (SCH‐3540MD Pulse magnetizer, Shanghai Pingye Co Ltd, China). Then, the magnetic patch was located on a cantilever beam. The entire structure was encapsulated in PDMS, which was cured at 60 °C for 120 min to package the sensor.

### Mechanical Characterization

Mechanical tests of the Polyimide, Polystyrene, and Polyurethane substrates (thickness of each substrate: 100 µm; width: 1 cm; length: 3 cm; Figure S9, Supporting Information) were conducted by using a computer‐controlled material testing machine (Instron 5943, USA) at the rate of 5 mm min^−1^. Stretching experiments were done using a laboratory‐prepared tensile test machine.

### Morphological Characterization

SEM images and EDS data of Co‐based amorphous wires were taken using microscope Sirion200 (FEI, USA).

### Magnetic Characterization

The hysteresis loop of the Co‐based amorphous wire was measured by applying a magnetic field along the wire in a vibrating sample magnetometer (Lakeshore7410, Lakeshore, USA). The MI of the amorphous wire was measured in an electromagnet while a Gauss meter was used to measure the magnitude of the magnetic field in the geometric center of the Helmholtz coil. The sensor's impedance during the current change was measured by the impedance analyzer (IM3570, HIOKI, Japan).

### Device Characterization

Sensor performance at small magnetic fields (0–1 mT, Figure 3b): The sensor was fixed in the center of the Helmholtz coil having 50 turns and 23 mm diameter. The magnetic field in the center of the coil was calculated as:

(3) $$B\;=\frac{\mu_{0}N_{0}IR^{2}}{{\left[R^{2}+{\left(\frac{d}{2}\right)}^{2}\right]}^{\frac{3}{2}}}$$

where μ_0_, *N*_0_, *I*, *R*, and *d* represent vacuum permeability, number of turns, current, spacing of two coils, and diameter of coil, respectively. The impedance change of the sensor with the change of the current through the coil was measured by using an impedance analyzer.

Sensor performance at strong magnetic fields (0.5 mT–400 mT, Figure 3c): The sensor was fixed in an electromagnet. A Gauss meter (PF‐35, Litian Co, Ltd., China) was used to measure the magnitude of the magnetic field in the centre between poleshoes of the electromagnet. An impedance analyzer was used to measure the impedance of the sensor induced due to magnetic field change by the current.

### Demonstrators

The sensor was fixed on the nail and an impedance analyzer (IM3570, HIOKI, Janpan) was used to measure the impedance change of the sensor. Data obtained from the impedance analyzer were processed using LabVIEW software. The angle between the sensor and the geomagnetic field direction changes upon the finger movement. This signal was used to control the movement of a car in a video game. A small patch of a flexible magnet (0.5 cm × 1 cm) was fixed on the arm with a PU tape. When the finger approaches a progressively larger electromagnet (threshold set at 200 mT), the software was programmed to issue a warning signal.

## Conflict of Interest

The authors declare no conflict of interest.

## Supporting information

Supporting Information

Supplemental Video 1

Supplemental Video 2

## Data Availability

The data that support the findings of this study are available from the corresponding author upon reasonable request.
