# Supplementary material for: Wearable Magnetic Field Sensor with Low Detection Limit and Wide Operation Range for Electronic Skin Applications
Source: Adv Sci (Weinh). 2023 Nov 30;11(37):2304525. doi: 10.1002/advs.202304525 (PMC11462294; doi:10.1002/advs.202304525)
Supplement: Supplementary file 1 — Supporting Information [file ADVS-11-2304525-s002.pdf]

ADVANCED SCIENCE
Open Access

Supporting Information

for *Adv. Sci.*, DOI 10.1002/advs.202304525

Wearable Magnetic Field Sensor with Low Detection Limit and Wide Operation Range for Electronic Skin Applications

Shengbin Li, Yuanzhao Wu, Waqas Asghar, Fali Li, Ye Zhang, Zidong He, Jinyun Liu, Yuwei Wang, Meiyong Liao, Jie Shang, Long Ren, Yi Du, Denys Makarov*, Yiwei Liu* and Run-Wei Li**

© Copyright 2020. WILEY-VCH GmbH.

Supporting Information

Wearable Magnetic Field Sensor with Low Detection Limit and Wide Operation Range for Electronic Skin Applications

Shengbin Li, Yuanzhao Wu, Waqas Asghar, Fali Li, Ye Zhang, Zidong He, Jinyun Liu, Yuwei Wang, Meiyong Liao, Jie Shang, Long Ren, Yi Du, Denys Makarov*, Yiwei Liu*, Run-Wei Li**

Supporting movies:

Video S1. Detection of the geomagnetic field to control the game using wearable magnetic field sensor. The sensor is integrated in a nail. When the finger moves, we rely on the change of the magnetic impedance of the sensor to control the movement of a car in the game.

Video S2. Warning and safety protection when a person is exposed to a strong magnetic field. The wearable magnetic field sensor is integrated in a fingernail. When the finger approaches an electromagnet with a strong magnetic field (here, stronger than 100 mT), the sensor triggers a warning signal.

Supporting Table:

Table S1. Comparison table of the flexible magnetic sensor with literature data.

Type	Materials	Substrate	Max Range (mT)	Min Range (mT)	Ref.
GMR	50 Å Ta/85 Å Py/22.5 Å Cu/32 Å Py/100 Å Fe46Mn54/10 Å Cu	Mylar/Transpa rency/Kapton polyimide	4	N/A.	[1]
GMR	IrMn (10 nm)/FeCo (4 nm)/Cu (3 nm)/FeCo (1 nm)/FeNi (6 nm)/FeCo (1 nm)/Cu (3 nm)/FeCo (4 nm)/IrMn (10 nm) and Ta buffer/capping layers	PDMS	90	N/A	[2]
GMR	[Py/CoFe]/Cu/[CoFe/Py]/IrMn het erostructure	Polyimide foils	40	2.0E0	[3]
GMR	Co/Cu flakes	Polyimide foils	600	N/A	[4]
GMR	$Ni_{81}Fe_{19}$(Py)/Cu	Si wafers	50	N/A	[5]
GMR	Ta (2 nm)/IrMn (5 nm)/[Py (4 nm)/CoFe (1 nm)]/Cu (1.8 nm)/[CoFe (1 nm)/Py (4 nm)	PDMS	30	1.2E0	[6]
TMR	Co/Al_2O_3 /Co	Flexible Gel-film®	10	N/A	[7]
TMR	The magnetic tunnel junction (MTJ) using MgO barrier	Ultrathin flexible silicon membrane	60	N/A	[8]
AMR	Permalloy (Py) Hall crosses	PET foils	0.05	2.0E-4	[9]
AMR	$Ni_{81}Fe_{19}$(Py)	PET foils	3	1.5E-4	[10]
AMR	Py/Ta powder	Mylar foils	400	6.0E-2	[11]
Magnetically driven stress	Carbonyl-iron µPs in PDMS	N/A	150	N/A	[12]
Magnetically driven stress	PDMS/Fe_3O_4 composites and flexible organic tribotronic transistor	PET foils	150	1.0E0	[13]
Magnetically driven stress	AgNWs–Fe_3O_4–PDMS and flexible organic transistor	Polyimide foils	150	5.0E-1	[14]
LMR	Bi	Polyimide foils	5000	1.4E-2	[15]
Hall	Graphene	Flexible Kapton foil	18	N/A	[16]
**GMI**	**Co-based Amorphous wire**	**Polyimide foils**	**400**	**2.2E-5**	**This work**

Supporting Figures:

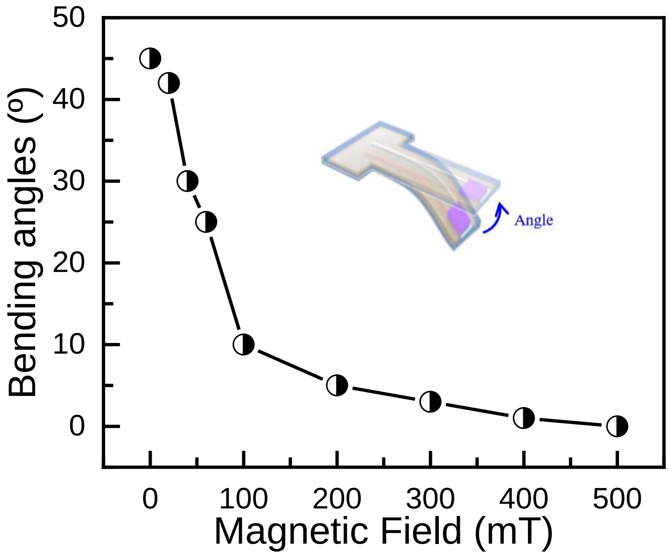

Figure S1. Bending angle of cantilever beam under different magnetic fields.

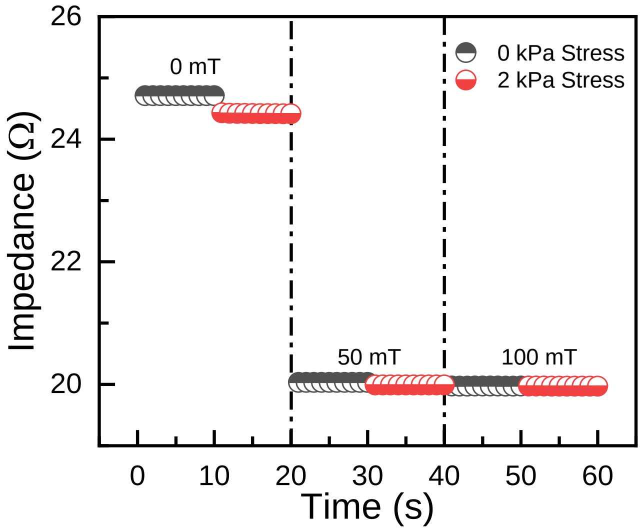

Figure S2. Changes of the impedance of the Co-based amorphous wire in an applied magnetic field with 3 MHz driving frequency and 1 mA driving current. The wire is exposed to mechanical pressure.

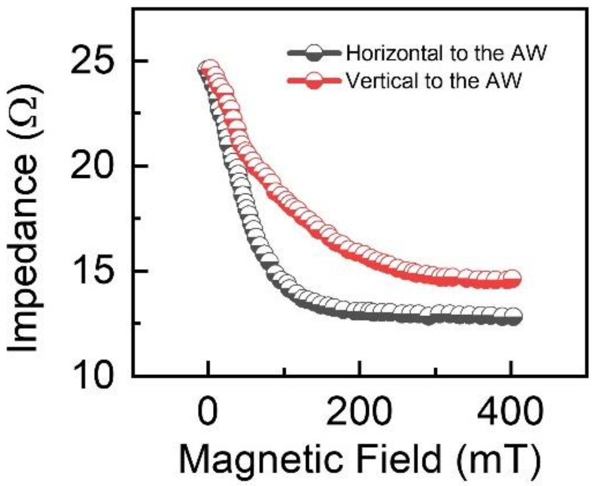

Figure S3. Variation of the impedance of the Co-based amorphous wire (AW). The wire is exposed to an external magnetic field applied along the wire (indicated as "horizontal") or perpendicular to the wire (indicated as "vertical").

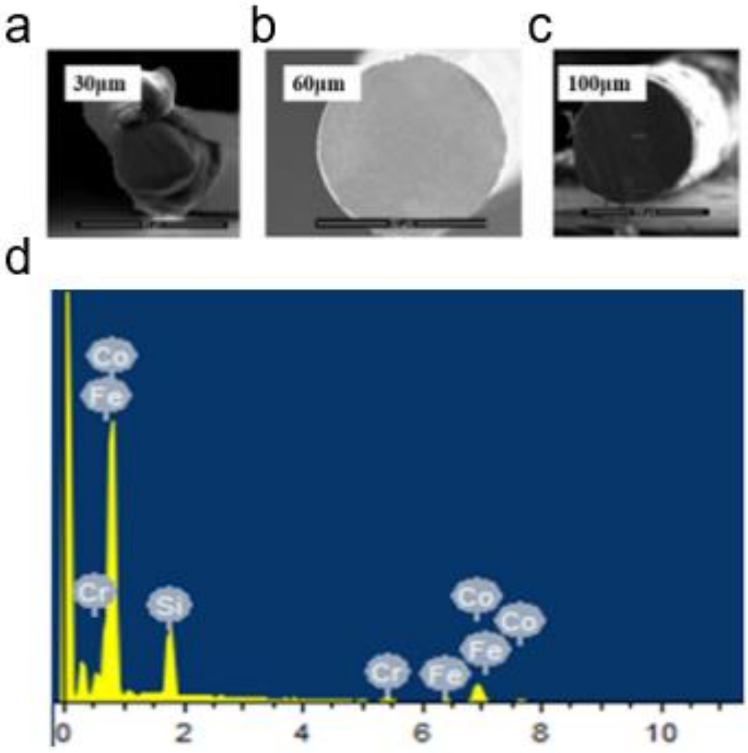

Figure S4. Microscopic images and elemental compositions of amorphous wires with different diameters: (a) SEM images of amorphous wires, (b) Electron dispersion spectrum of amorphous wires.

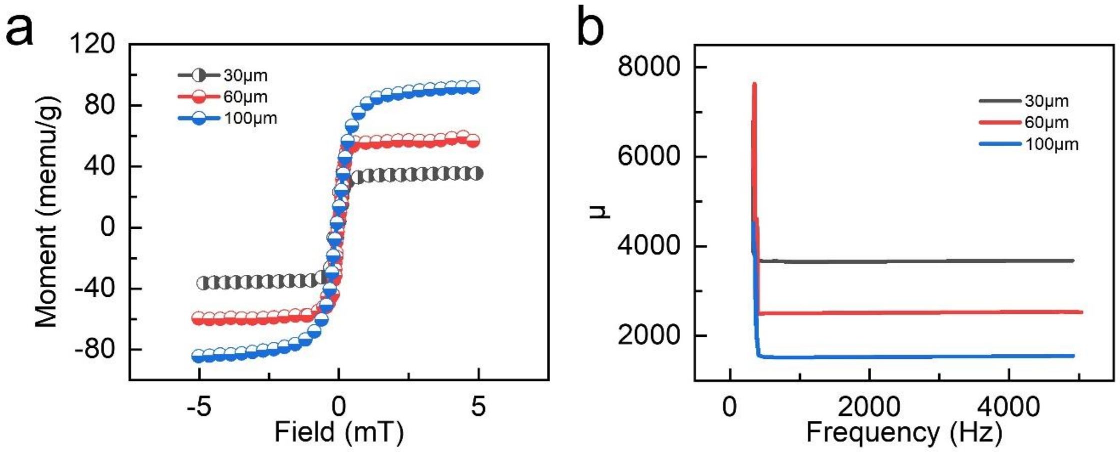

Figure S5. Magnetic properties of amorphous wires with different diameters: (a) Hysteresis loops of amorphous wires with diameters of 30, 60, 100 microns. (b) Magnetic permeability of amorphous wires with diameters of 30,60,100 microns.

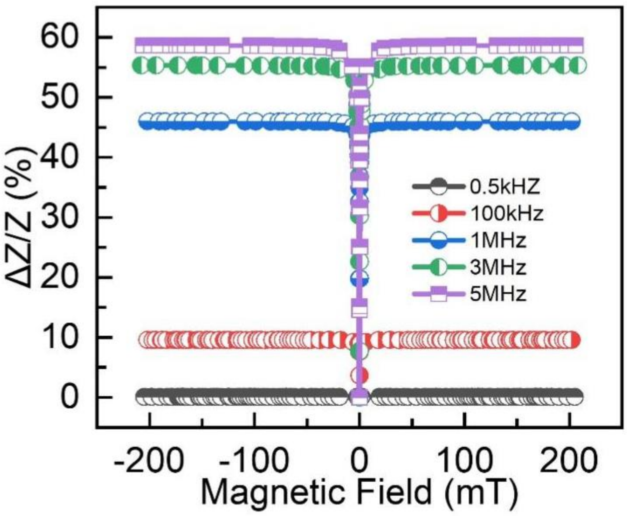

Figure S6. Variation of the impedance of the amorphous wire with diameter of 30 µm in an applied magnetic field. The measurement is conducted when exciting the wire at different frequencies.

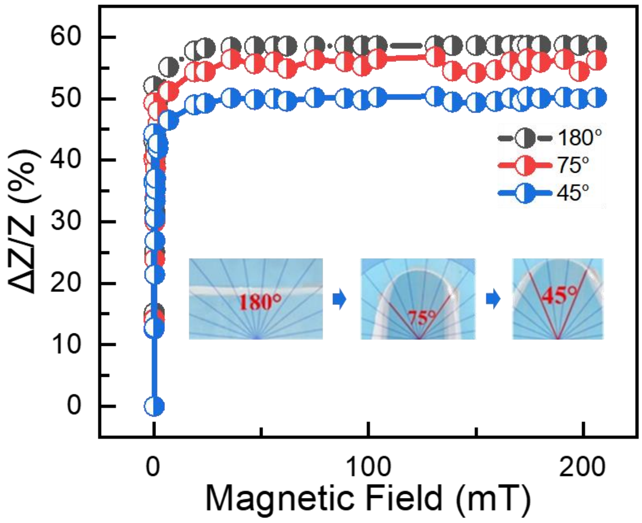

Figure S7. Response curves of amorphous wire to magnetic field at different angles. Change measurement of the impedance with the magnetic field when the amorphous wire is bent at 180 degrees (no bent), 90 degrees, and 45 degrees, with a driving current of 1mA and a frequency of 5MHz.

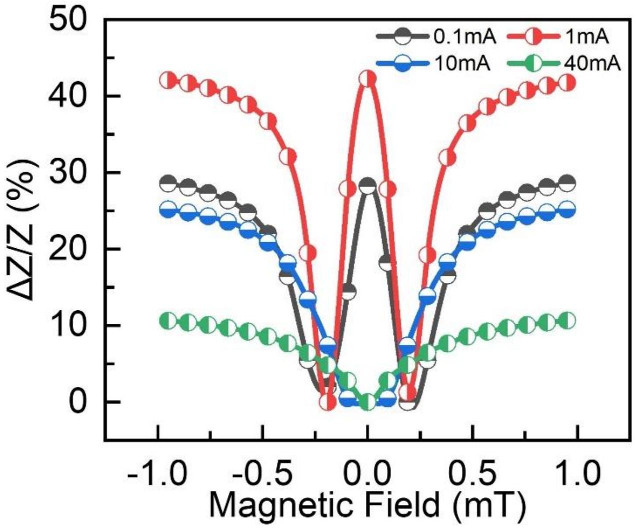

Figure S8. MI performance of the sensor under different driving currents.

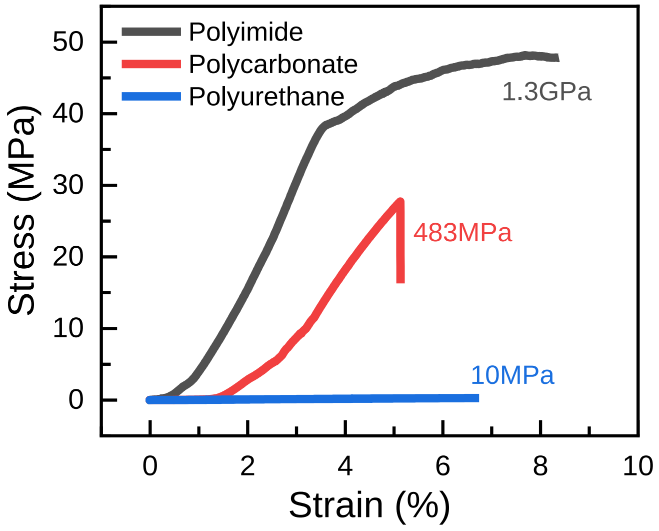

Figure S9. Stress-strain curves of different substrates. The thickness of each substrate is 0.1 mm, the width and length of the tested sample is 1 cm and 3 cm respectively, and the rate of applying strain is 0.5%/s.

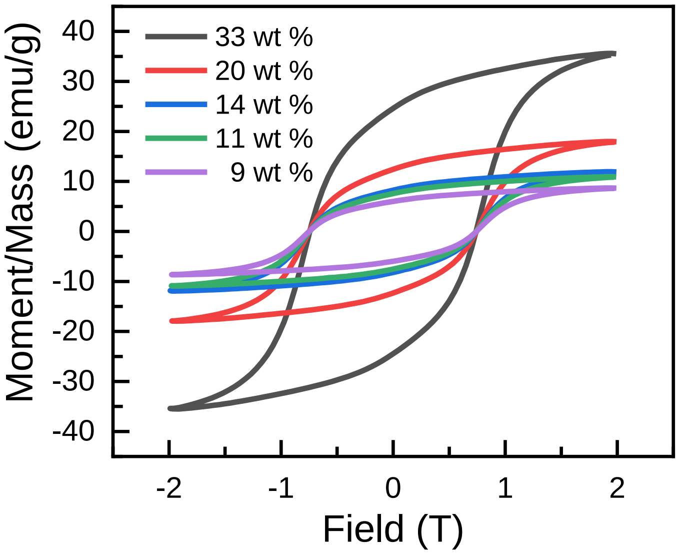

Figure S10. Hysteresis loops of flexible magnets with different NdFeB wight ratios.

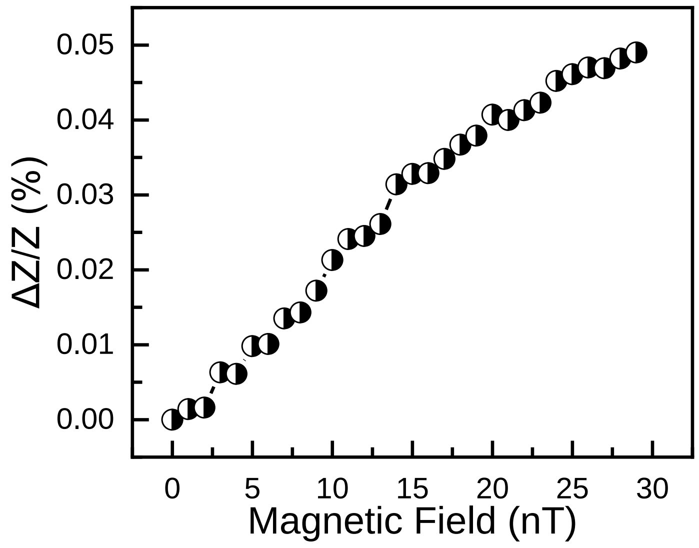

Figure S11. Sensor response to magnetic field from 0 to 30 nT.

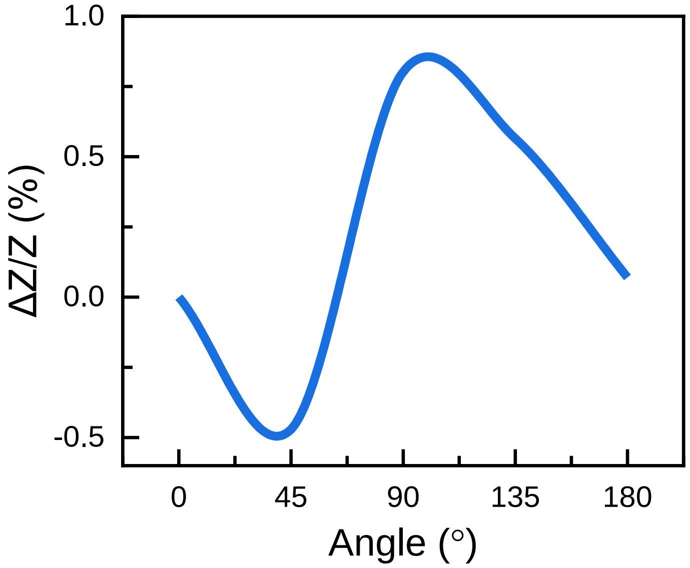

Figure S12. Hysteresis loops of flexible magnets with different NdFeB wight ratios.

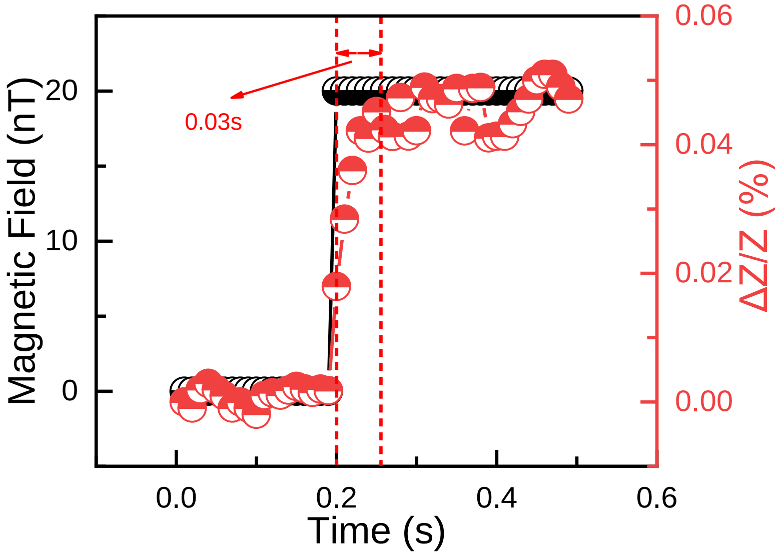

Figure S13. The response time of the magnetic field after the sensor applies 22nT.

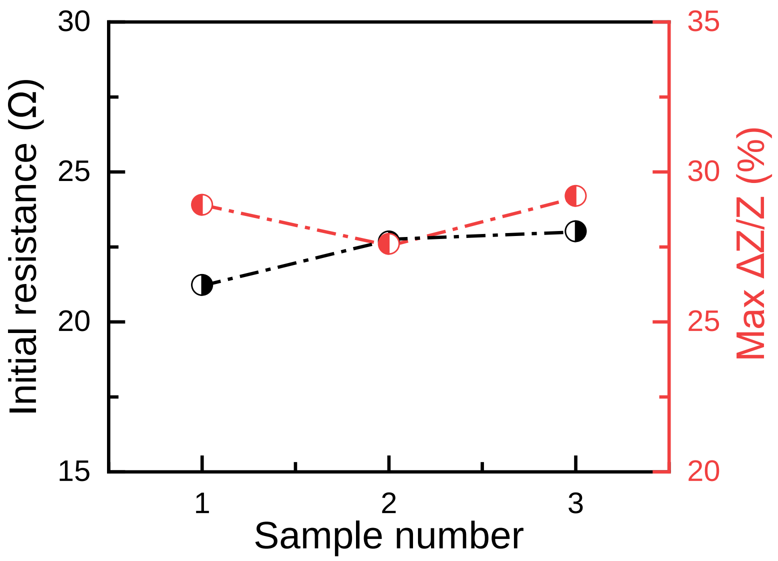

Figure S14. Initial impedance and maximum impedance change rate under saturation for three sensors fabricated repeatedly.

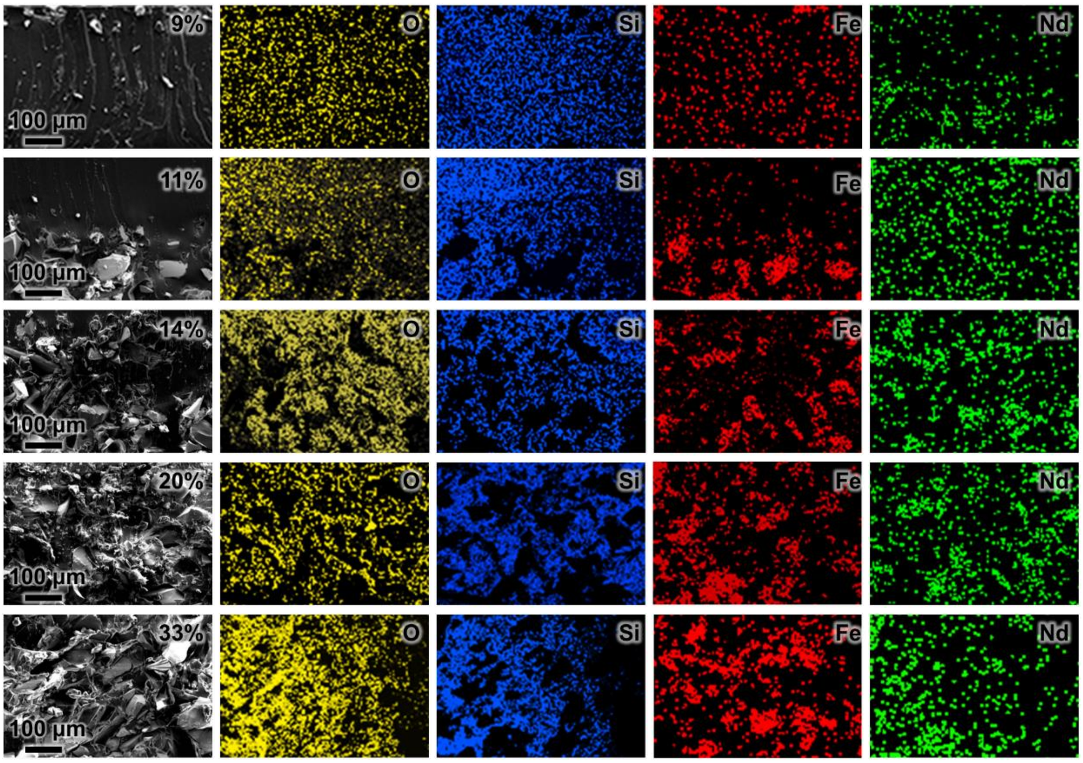

Figure S15. Scanning electron microscope and element distribution of NdFeB with different wight ratios (9 wt%, 11 wt%, 14 wt%, 20 wt%, 33 wt%).

References

[1] S. S. P. Parkin, Appl. Phys. Lett. 1996, 69, 3092.
[2] H. Li, Q. Zhan, Y. Liu, L. Liu, H. Yang, Z. Zuo, T. Shang, B. Wang, R. W. Li, ACS Nano 2016, 10, 4403.
[3] G. S. Canon Bermudez, D. D. Karnaushenko, D. Karnaushenko, A. Lebanov, L. Bischoff, M. Kaltenbrunner, J. Fassbender, O. G. Schmidt, D. Makarov, Sci. Adv. 2018, 4, eaao2623.
[4] D. Karnaushenko, D. Makarov, M. Stober, D. D. Karnaushenko, S. Baunack, O. G. Schmidt, Adv. Mater. 2015, 27, 880.
[5] N. Pérez, M. Melzer, D. Makarov, O. Ueberschär, R. Ecke, S. E. Schulz, O. G. Schmidt, Appl. Phys. Lett. 2015, 106, 153501.
[6] M. Melzer, G. Lin, D. Makarov, O. G. Schmidt, Adv. Mater. 2012, 24, 6468.
[7] C. Barraud, C. Deranlot, P. Seneor, R. Mattana, B. Dlubak, S. Fusil, K. Bouzehouane, D. Deneuve, F. Petroff, A. Fert, Appl. Phys. Lett. 2010, 96, 072502.
[8] J. Y. Chen, Y. C. Lau, J. M. Coey, M. Li, J. P. Wang, Sci. Rep. 2017, 7, 42001.
[9] P. N. Granell, G. Wang, G. S. Cañon Bermudez, T. Kosub, F. Golmar, L. Steren, J. Fassbender, D. Makarov, npj Flexible Electron. 2019, 3, 3.
[10] Z. Wang, X. Wang, M. Li, Y. Gao, Z. Hu, T. Nan, X. Liang, H. Chen, J. Yang, S. Cash, N. X. Sun, Adv. Mater. 2016, 28, 9370.
[11] E. S. Oliveros Mata, G. S. Cañón Bermúdez, M. Ha, T. Kosub, Y. Zabila, J. Fassbender, D. Makarov, Appl. Phys. A 2021, 127, 280.
[12] L. Ding, S. Xuan, L. Pei, S. Wang, T. Hu, S. Zhang, X. Gong, ACS Appl. Mater. Interfaces 2018, 10, 30774.
[13] J. Zhao, H. Guo, Y. K. Pang, F. Xi, Z. W. Yang, G. Liu, T. Guo, G. Dong, C. Zhang, Z. L. Wang, ACS Nano 2017, 11, 11566.
[14] Y. Zang, F. Zhang, D. Huang, C. A. Di, D. Zhu, Adv. Mater. 2015, 27, 7979.
[15] E. S. Oliveros-Mata, C. Voigt, G. S. Cañón Bermúdez, Y. Zabila, N. M. Valdez-Garduño, M. Fritsch, S. Mosch, M. Kusnezoff, J. Fassbender, M. Vinnichenko, D. Makarov, Adv. Mater. Technol. 2022, 7, 2200227.
[16] Z. Wang, M. Shaygan, M. Otto, D. Schall, D. Neumaier, Nanoscale 2016, 8, 7683.
